# Lard as an Antidote for Strychnine-poisoning

**Published:** 1898-07

**Authors:** W. D. Turner


### LARD AS AN ANTIDOTE FOR STRYCHNINE-POISONING.

By W. D. Turner, M.D.

I wish to give the results of a series of experiments made with the sulphate of strychnine upon the lower animals, and to suggest an antidote for the same.

The way my attention was first called to this treatment was as follows: A very valuable dog accidentally ate some strychnine placed upon a piece of meat for other purposes, and when found was in the agony of death, it seemed. Where he had been lying on the ground he had (from convulsions) scratched a hole larger than he was; he must have eaten the strychnine several hours ahead of the time he was found. He was so very near dead that I thought it useless to attempt to do anything; but one of the employés on my place, to be doing something, gave him lard—I guess about four ounces, as near as I can judge from what he told me. In less than five hours the dog was up running about, and the next day seemed as well as ever. Then I commenced my investigations or experiments.

I took a full-grown dog and gave him three grains of the sulphate of strychnine and waited till the spasms were well advanced, which was in about twenty-eight minutes; waited forty-eight minutes and gave six ounces of lard. In two and one-half hours he was up, though for two days was very stiff and sore. Three days thereafter I gave the same dog four grains of the sulphate of strychnine, but the convulsions were not well advanced until about thirty-five minutes. I waited an hour before I gave the lard, six ounces, and had to repeat it in an hour. In thirty-five minutes from the second dose he was up. This is the only instance in which I used it on the same animal the second time.

This, the last dog experimented upon, was given four grains of the sulphate of strychnine. I waited until his legs were fixed and stiff, and had to force his jaws open to pour the lard down him; I had to wrap him in warm cloths, and keep warm water applied to the surface to retain normal temperature. I gave him eighteen ounces of lard and kept him warm, and in twelve hours he was up, though he would not eat till the next day.

I next gave a hen one-quarter grain of strychnine sulphate, but the only effect it had was to make her drunk. Gave one teaspoonful of melted lard, and the next morning she was going about, though not eating. I had about the same experience with two other hens.

I next tried one-fourth grain on a crow, and in twenty-four hours it flew away, after using the lard.

I next tried it on four eight-months-old pigs; I lost the first two, but gave twelve ounces to the last two, instead of six ounces, and they both got well. I only gave two grains of strychnine sulphate to each hog.

I then gave an eleven-months-old calf six grains of strychnine sulphate, and with fifteen ounces of lard had good results in six hours, though I did not let the convulsions become very marked before I gave the lard, as it was a very valuable calf, and I could not well afford to lose it; but with the next calf I gave a good test.

The next was a six-months-old calf, to which I gave five grains of strychnine sulphate. In forty minutes the convulsions were well-marked; I then gave fifteen ounces of lard, but in an hour had to give seven ounces more. Made a good recovery.

I have tried to be very brief, and have sacrificed many points and much grammar for the sake of brevity; but I did wish to let these little experiments be known, and let them be carried out further, if deemed worthy, for it does seem to me that in lard we have a safe, sure, and simple antidote for strychnine-poisoning.—*Virginia Medical Semi-Monthly.*

